# An allele of rs619586 polymorphism in MALAT1 alters the invasiveness of meningioma via modulating the expression of collagen type V alpha (COL5A1)

**DOI:** 10.1111/jcmm.15637

**Published:** 2020-07-28

**Authors:** Jie Zheng, Chang‐he Pang, Wei Du, Lei Wang, Lai‐guang Sun, Zhen‐yi Xing

**Affiliations:** ^1^ Department of Neurosurgery Xinxiang Central Hospital Xinxiang China; ^2^ Department of Neurosurgery The First Affiliated Hospital of Zhengzhou University Zhengzhou China

**Keywords:** COL5A1, MALAT1, meningioma, MiR‐145, polymorphism, Rs619586

## Abstract

The rs619586 polymorphism has been shown to alter the expression of MALAT1, which act as a competing endogenous RNA (ceRNA) against miR‐145. And miR‐145 was found to target COL5A1, the interaction between which was shown to be involved in the pathogenesis of invasive meningioma. In this study, we aimed to explore the effect of rs619586 polymorphism and its underlying molecular mechanism in invasive meningioma. Real‐time PCR and Western Blot analysis were used to study the differentiated expression of miR‐145, MALAT1 (metastasis‐associated lung adenocarcinoma transcript 1) and COL5A1 (collagen alpha‐1(V) chain) in tumour/serum samples genotyped as rs619586 AA, AG and GG. Computational analysis and luciferase reporter assay were also conducted to identify the regulatory relationship between miR‐145 and MALAT1/COL5A1. Meanwhile, expression of miR‐145 and COL5A1 in different cell treatment groups was measured to validate the results obtained from earlier experiments. As shown by the results and in tumour/serum samples genotyped as AA, AG and GG, the expression of both MALAT1 and COL5A1 was down‐regulated in a stepwise fashion, while the expression of miR‐145 was increased, suggesting a potential negative relationship between MALAT1/COL5A1 and miR‐145. Meanwhile, miR‐145 was shown to bind to MALAT1, while COL5A1 was identified as a virtual target gene of miR‐145. As a consequence, a MALAT1/miR‐145/COL5A1 molecular pathway was established based on the above results. In particular, with the presence of rs619586 A>G polymorphism, the expression of MALAT1 and COL5A1 was both reduced, leading to reduced invasiveness of meningioma.

## 1 INTRODUCTION

Meningioma is a type of intracranial tumour with a high incidence in adulthood.^1^ While meningioma usually has a good prognosis after a surgical operation, some meningioma patients are associated with a higher risk of recurrence and a poorer prognosis.^2^ Penetration of meningioma into adjacent brain parenchyma is an important factor associated with its recurrence and leads to even poorer prognosis.^3^ Although meningioma has benign characteristics in general, brain invasive meningiomas have been considered as grade II tumours by the World Health Organization (WHO).^2^

Ranging in size from 200 nt to >100 kb, long noncoding RNAs (lncRNAs) are the most complex and the largest family of noncoding RNAs (ncRNAs). In addition, the expression of many lncRNAs is specific to cell types. The expression of a majority of lncRNAs is quite low, with some only expressed at one copy per cell.^4^ However, lncRNAs are involved in various molecular functions, such as regulating protein activities, controlling transcriptional activities, playing organizational or structural roles, modifying RNA processing or acting as small RNA precursors.^5^

As an lncRNA exerting oncogenic effects in many cancers, MALAT1 participates in a wide range of cellular processes.^6^ For example, MALAT1 has been shown to regulate the expression of miR‐145, a molecule related to cardiomyocyte injury.^7^ Moreover, miR‐145 expression in HL‐1 cells was negatively regulated by MALAT1. Furthermore, the expression of BCL2/adenovirus E1B 19 kD protein‐interacting protein 3 (Bnip3), a target gene of miR‐145, is reduced with the decreasing miR‐145 expression.^8^

It was demonstrated that the reduction in miR‐145 expression during aggressive meningiomas is related to increased cancer invasiveness and motility, while only minor reduction in the expression of miR‐145 targets has been observed, with COL5A1 most significantly down‐regulated in miR‐145‐positive cells. Furthermore, in vitro studies also detected a higher level of COL5A1 mRNA in primary meningioma showing poor miR‐145 expression.^9^

It was found that the rs619586A> G single nucleotide polymorphisms (SNP) provided a protective effect on pulmonary arterial hypertension (PAH). Other functional bioassays also showed that the change from rs619586A to G in MALAT1 could promote its role as a competing endogenous RNA (ceRNA) of miR‐214 and consequentially up‐regulate the expression of X‐box binding protein 1 (XBP1), thus suppressing the migration and proliferation of vascular endothelial cells. In addition, it was shown that the rs619586A> G SNP in MALAT1 was related to a decreased risk of PAH. Animal experiments also indicated that the alteration from rs619586 A to G affected the function of MALAT1 in accelerating cell migration and proliferation.

The rs619586 polymorphism has been shown to alter the expression of MALAT1.^10^ In addition, MALAT1 was found to act as a competing endogenous RNA (ceRNA) against miR‐145, while COL5A1 was found to be a target of miR‐145. In fact, the deregulation of both miR‐145 and COL5A1 was shown to be involved in the pathogenesis of invasive meningioma.^9^, ^11^ In this study, we studied the effect of rs619586 on the signalling pathway of MALAT1/miR‐145/COL5A1 and investigated the association between rs619586 polymorphism and the invasiveness of meningioma.

## 2 MATERIALS AND METHODS

### 2.1 Human subjects and sample collection

A total of 829 patients diagnosed with meningioma by histological examination and treated by radical resection at our institute were enrolled in this study. These patients were divided to two groups, that is, an invasive meningioma group (N = 427) and a non‐invasive meningioma group (N = 402) based on the invasiveness of their malignancy. Invasiveness of meningioma is defined as those meningioma that is invasive into the neighbouring brain parenchyma and evaluated by imaging tests. The serum samples were collected from all patients and were immediately frozen in liquid nitrogen at −80°C after collection. In addition, among all these 427 patients enrolled at the beginning of our study, we further selected 74 invasive meningioma patients according to the inclusion criteria listed as below: 1. no history of other malignant tumour; 2. primary meningioma; 3. no history of chemotherapy, radiotherapy or other treatment before the operation described in this study; 4. all sample sections examined by at least 2 pathologists were consistent with the diagnostic criteria of meningioma; 5. with complete clinicopathological and follow‐up data. Accordingly, the rs619586 polymorphism in 74 patients was genotyped and the patients were divided to the following groups according to their rs619586 polymorphism genotypes: AA (N = 42), AG (N = 25) and GG (N = 7). We collected surgically resected meningioma and serum samples from these patients for subsequent analyses. This study was approved by the Institutional Review Board of our institute, and written informed consent was obtained from all patients.

### 2.2 Genotyping by Taqman assay

Genotyping of rs619586 polymorphism was conducted using a TaqMan assay (Applied Biosystems, Foster City, CA). In brief, the reactions between the TaqMan probes and PCR primers were carried out in a 96‐well plate on a thermal cycler. Subsequently, the intensity of fluorescence was detected on an ABI 7500 qPCR machine (Applied Biosystems, Foster City, CA) and analysed using the manufacturer's software.

### 2.3 RNA isolation and real‐time PCR

Total RNA was extracted from tissues using a Trizol Kit (Invitrogen). The optical density (OD) value (at 260 nm and 280 nm) and RNA concentration were measured on a nucleic acid/protein analyzer (BioPhotometer D30, Eppendorf, Hamburg, Germany). An OD260 nm/OD280 nm ratio of between 1.8 and 2.0 was considered as high purity. Reverse transcription was performed using a PrimeScript RT Kit (Fermentas). The reaction conditions were as follows: 70°C for 5 minutes, 4°C for 3 minutes, 37°C for 60 minutes, and 95°C for 10 minutes. The primer sequences for MALAT1, miR‐145, COL5A1 mRNA and internal control glyceraldehyde‐3‐phosphate dehydrogenase (GAPDH) were designed and synthesized by Genechem. Fluorescent quantitative PCR was performed with a SYBR^®^ Premix Ex TaqTM II Kit (Xingzhi Biotech). The reaction system consisted of 5.3 μL 2× Taq Master Mix, 1 μL forward primer (5 µmol/L), 1 μL reverse primer (5 µmol/L), 1 μL cDNA and 11.7 μL RNase Free H_2_O. The PCR reaction conditions were as follows: pre‐denaturation at 95°C for 30 seconds and 35 cycles of denaturation at 94°C for 15 seconds, annealing at 56°C for 45 seconds and extension at 72°C for 45 seconds. The gene expression was detected on an ABI 7500 qPCR machine. Using GAPDH as an internal control, the relative expression of target genes was calculated based on the 2^−ΔΔCt^ method, in which ΔΔCt = (the mean Ct value of target gene in the experimental group−the mean Ct value of house‐keeping gene in the experimental group)−(the mean Ct value of target gene in the control group−the mean Ct value of house‐keeping gene in the control group). Each measurement was repeated three times.

### 2.4 Cell culture and transfection

KNS‐89 and SNB‐19 cells were purchased from Institute of Biochemistry and Cell Biology, Chinese Academy of Sciences. The cells were cultured in a DMEM culture medium (Invitrogen) supplemented with 10% foetal bovine serum (FBS), 100 U/mL penicillin and 100 mg/mL streptomycin. The cells were incubated in an incubator under saturated humidity, 5% CO_2_ and 37°C. The medium was replaced every 1 or 2 days according to the status of cell growth. Sub‐culture was conducted when cell confluence reached 80%‐90%. When the cell confluence reached 70%, the medium was replaced by a serum‐free medium and the cells were seeded in a 6‐well plate at 24 hours before transfection. When the cells reached 50% confluence, they were transfected with MALAT1, miR‐145 mimics, anti‐miR‐145 or a miR‐145 negative control using Lipofectamine 2000 (Invitrogen) according to the manufacturer's protocol. After transfection, the cells were cultured at 37°C with 5% CO_2_ for 18 hours. The medium was then replaced with a complete medium, and the cells were collected 48 hours after transfection. Each measurement was repeated three times.

### 2.5 Vector construction and mutagenesis

The putative miR‐145 binding site within the 3′‐UTR of MALAT1 was identified using TargetScan (targetscan.org). Subsequently, the 3′‐UTR of the MALAT1 gene was amplified by PCR, followed by sub‐cloning the PCR product into a pcDNA Luciferase Reporter Vector (Promega, Madison, WI) to generate a luciferase construct containing the 3′ untranslated region (3′UTR) of wild‐type MALAT1. Meanwhile, the binding site of miR‐145 located in the 3′UTR of MALAT1 was mutated using a QuikChange Lightening Site‐Directed Mutagenesis Kit (Stratagene), and the mutant 3′UTR of MALAT1 was inserted to another pcDNA Luciferase Reporter Vector to generate a luciferase construct containing the mutant 3′UTR of MALAT1. Similarly, to study the effect of miR‐145 silencing, an anti‐miR‐145 sequence was cloned into pcDNA Luciferase Reporter Vector to generate pcDNA‐anti‐miR‐145.

### 2.6 Luciferase assay

After KNS‐89 and SNB‐19 cells were co‐transfected for 48 hours with wild‐type or mutant MALAT1 and miR‐145 mimics or a negative control, the luciferase activity in transfected cells was measured using a luciferase assay kit (Genecopoeia) in conjunction with a Glomax 20/20 luminometer (Promega). Similarly, KNS‐89 and SNB‐19 cells were co‐transfected with wild‐type or mutant COL5A1 and miR‐145 mimics or a negative control, and the luciferase activity in transfected cells was measured at 48 hours after transfection. Each experiment was repeated three times.

### 2.7 Western blot analysis

After being washed twice by PBS, meningioma tissue samples were ground in liquid nitrogen and made into homogenate with the addition of a lysis buffer. Subsequently, sample tissues were centrifuged at 1200 r/min for 30 minutes at 4°C to remove the tissue debris. The supernatant was collected to measure the total protein concentration according to the instructions of a bicinchoninic acid (BCA) kit. The protein sample (50 µg) was mixed with a sodium dodecyl sulphate (SDS) loading buffer and boiled at 100°C for 5 minutes. In the next step, the protein was separated by 10% sodium dodecyl sulphate polyacrylamide gel (SDS‐PAGE) electrophoresis and transferred onto a polyvinylidene fluoride (PVDF) membrane by a wet spinning method. The membrane was then blocked with 5% skimmed milk for 1 hour at room temperature and then incubated overnight at 4°C with diluted (1:100) anti‐COL5A1 anti‐GAPDH (internal control) primary antibodies (Abcam, Cambridge, MA). Afterwards, the membrane was washed 3 times by Tris (‐HCL)‐buffered saline + Polysorbate 20 (Tween 20) (TBST) and further incubated for 1 hour with horseradish peroxidase (HRP)‐labelled secondary antibodies. In the next step, the membrane was soaked in an enhanced chemiluminescence (ECL) reagent (Biomiga) and photographed on an X‐ray apparatus (Qcbio). The ratio between the mean optical density (OD) value of COL5A1 and the mean OD value of GAPDH was calculated as the relative protein expression of COL5A1. Each measurement was repeated three times.

### 2.8 Statistical analysis

Statistical analysis was carried out using the SPSS 21.0 software (IBM). Data were presented as mean ± standard deviations. Differences between two groups were compared by t test, while differences among multiple groups were compared by one‐way analysis of variance (ANOVA). A *P* value of <.05 was considered statistically significant.

## 3 RESULTS

### 3.1 Rs619586 polymorphism is associated with the risk of meningioma

At the beginning of this study, we enrolled a total of 829 patients diagnosed with invasive meningioma (N = 427) or non‐invasive meningioma (N = 402) in this study, the demographic data of which were presented in Table 1. The genotype of rs619586 polymorphism was utilized as the basis to divide these meningioma patients for genotype and allele analysis, as presented in Table 2. In Table 2, we showed that heterozygote genotype (AG) is borderline associated with reduced risk of invasive meningioma, and the risk of invasive meningioma GG genotype is significantly lower compared with AA group. Meanwhile, the risk in AG+GG group was lower than the reference group (AA) for invasive meningioma patients. In addition, we also showed that G allele is significantly associated with the reduced risk of invasive meningioma.

**Table 1:** Demographic data of the participants

Characteristics	Invasive meningioma (N = 427)	Non‐invasive meningioma (N = 402)	*P* values
Age, mean (SD) year	55.6 (13.5)	53.9 (8.6)	.8364
Sex			
Male	163 (38.2)	157 (39.1)	.7942
Female	264 (61.8)	245 (60.9)	
Smoking status			
Never	318 (74.5)	293 (72.9)	.6040
Ever	109 (25.5)	109 (27.1)	
Drinking			
Never	308 (72.1)	278 (69.2)	.3466
Ever	119 (27.9)	124 (30.8)	
Family history of cancer			
Yes	83 (19.4)	81 (20.1)	.7973
No	344 (80.6)	321 (79.9)	
Location			
Convexity	158 (37.0)	132 (32.8)	.3127
Falcine	102 (23.8)	112 (27.8)	
Foramen magnum	21 (4.9)	25 (6.2)	
Parasagittal	24 (5.6)	32 (7.9)	
Petroclival region	18 (4.2)	24 (5.9)	
Sphenoid wing	89 (20.8)	72 (17.9)	
Suprasellar	15 (3.7)	5 (1.5)	

**Table 2:** Frequency of genotype and allele of rs619685 polymorphism in each group

Genotype	Invasive Meningioma (N = 427)	Non‐invasive Meningioma (N = 402)	OR (95% CI)	*P* values
Rs619586 genotype				
AA	240	184		
AG	153	157	1.33 (0.99‐1.79)	.052
GG	34	61	2.34 (1.47‐3.71)	<.001
AG+GG	187	218	1.52 (1.16‐1.99)	.003
Rs619586 allele				
A	633	525		
G	221	279	1.52 (1.23‐1.88	<.001

### 3.2 Possible negative relationship between MALAT1 and miR‐145 in tumour/serum samples of meningioma

**FIGURE 1:**
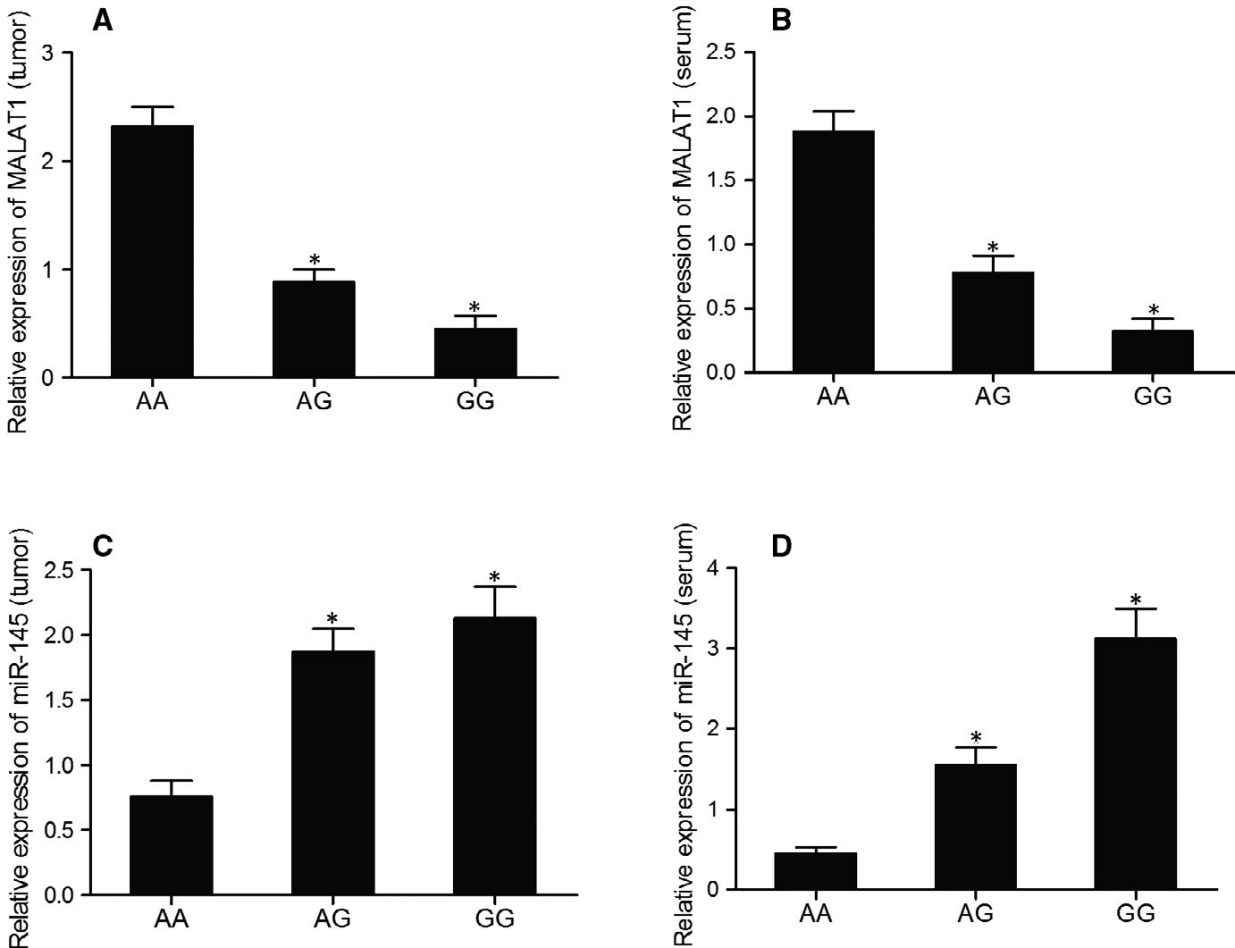
Differentiated expression of MALAT1 and miR‐145 in tumour/serum samples of various genotypes suggested a possible negative relationship between MALAT1 and miR‐145 (*P value < .05. vs AA group). A, Relative mRNA expression of MALAT1 in tumour samples of various genotypes; B, Relative mRNA expression of MALAT1 in serum samples of various genotypes; C, Relative mRNA expression of miR‐145 in tumour samples of various genotypes; D, Relative mRNA expression of miR‐145 in serum samples of various genotypes

Serum and tumour samples were collected from 74 invasive meningioma patients selected from 829 meningioma patients according to our inclusion criteria. Real‐time PCR was then conducted to detect the relative expression of MALAT1 and miR‐145 in these samples. As shown in Figure 1A, the relative mRNA expression of MALAT1 was the lowest in the samples genotyped as GG, the expression of MALAT1 was the highest in the samples genotyped as AA. The mRNA expression of MALAT1 in serum samples showed similar results (Figure 1B). Moreover, the mRNA expression of miR‐145 was the highest in the samples genotyped as GG and the lowest in the samples genotyped as AA (Figure 1C,D), suggesting a possible negative relationship between MALAT1 and miR‐145.

### 3.3 Differentiated expression of COL5A1

**FIGURE 2:**
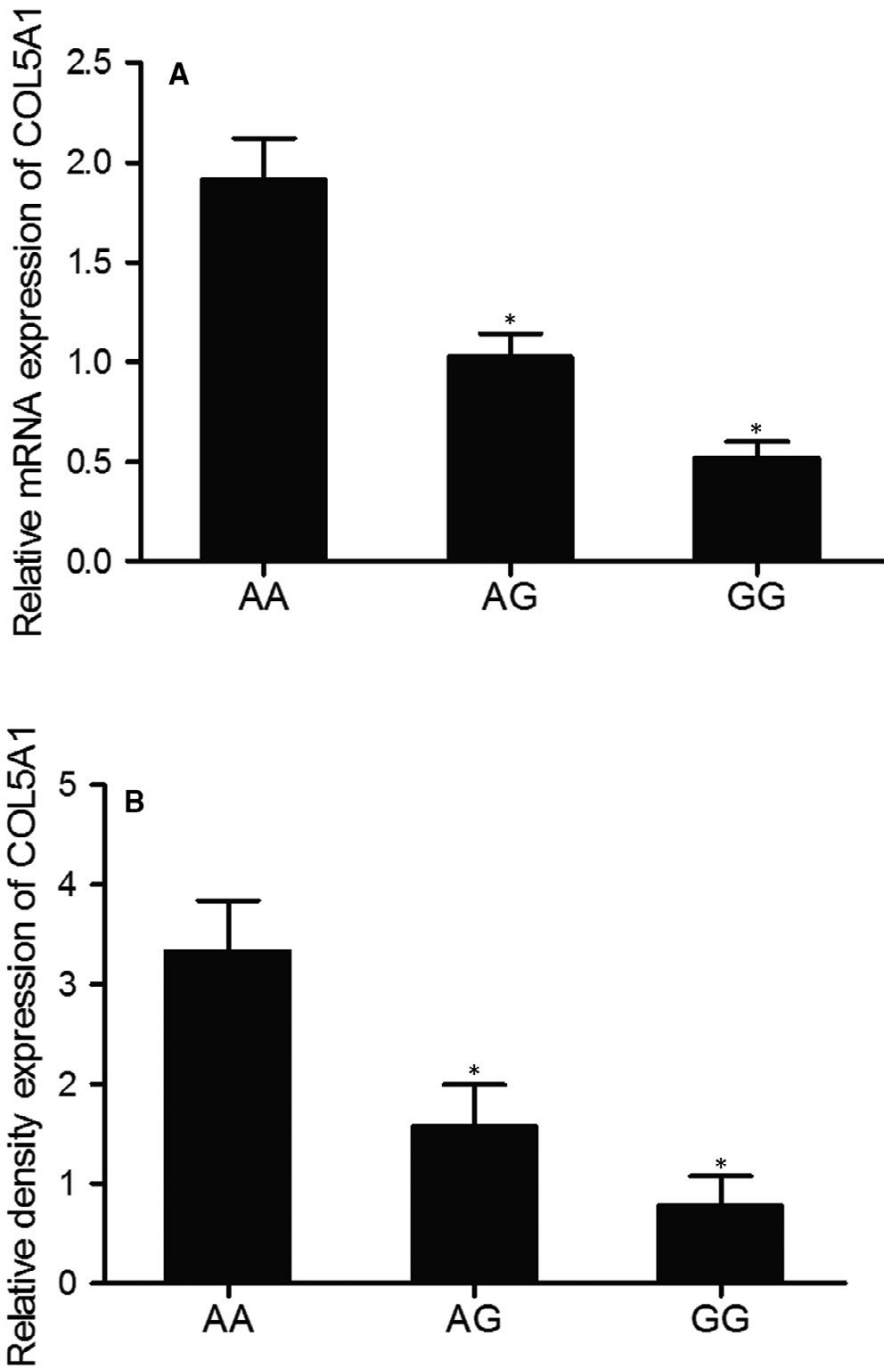
Expression of COL5A1 mRNA and protein in samples of various genotypes suggested a negative correlation between MALAT1 and miR‐145 (*P value < .05. vs. AA group). A, Relative mRNA expression of COL5A1 in samples of various genotypes; B, Relative protein expression of COL5A1 in samples of various genotypes

The expression of COL5A1 mRNA and protein was compared among AA, AG and GG groups using real‐time PCR and Western Blot assays. As shown in Figure 2A,B, the relative expression of both COL5A1 mRNA and protein was the lowest in the samples genotyped as GG and the highest in the samples genotyped as AA, suggesting a negative correlation between COL5A1 and miR‐145.

### 3.4 MiR‐145 was able to bind to MALAT1

**FIGURE 3:**
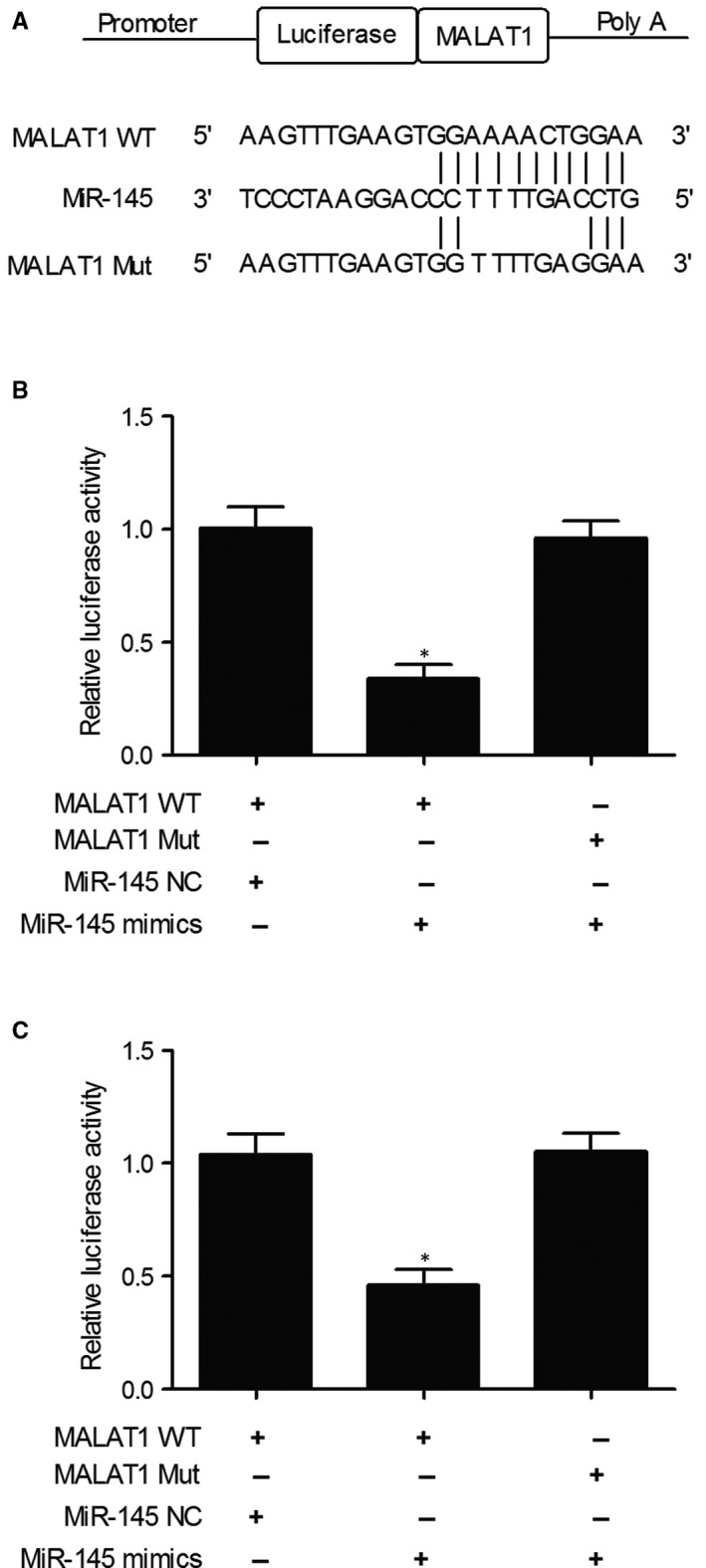
Computational analysis and luciferase assay showed a direct binding between miR‐145 and MALAT1 (*P value < .05. vs MALAT1 + miR‐145 NC group). A, Candidate seed sequences of miR‐145 in the 3′UTR of MALAT1 as detected by computational analysis; B, Luciferase activity of KNS‐89 cells transfected with wild‐type or mutant MALAT1 and miR‐145 mimics or miR‐145 negative controls; C, Luciferase activity of SNB‐19 cells transfected with wild‐type or mutant MALAT1 and miR‐145 mimics or miR‐145 negative controls

Computational analysis and luciferase assay were conducted to further explore the regulatory relationship between miR‐145 and MALAT1. As shown in Figure 3A, miR‐145 was able to bind to MALAT1 on its 3′UTR. Furthermore, the relative luciferase activity in KNS‐89 and SNB‐19 cells co‐transfected with the wild‐type MALAT1 and miR‐145 mimics was apparently reduced, while the relative luciferase activity in KNS‐89 and SNB‐19 cells co‐transfected with the mutant MALAT1 and miR‐145 mimics showed no significant difference (Figure 3), suggesting that miR‐145 acted as a direct inhibitor of MALAT1 by binding to its 3′UTR.

### 3.5 COL5A1 was a target gene of miR‐145

**FIGURE 4:**
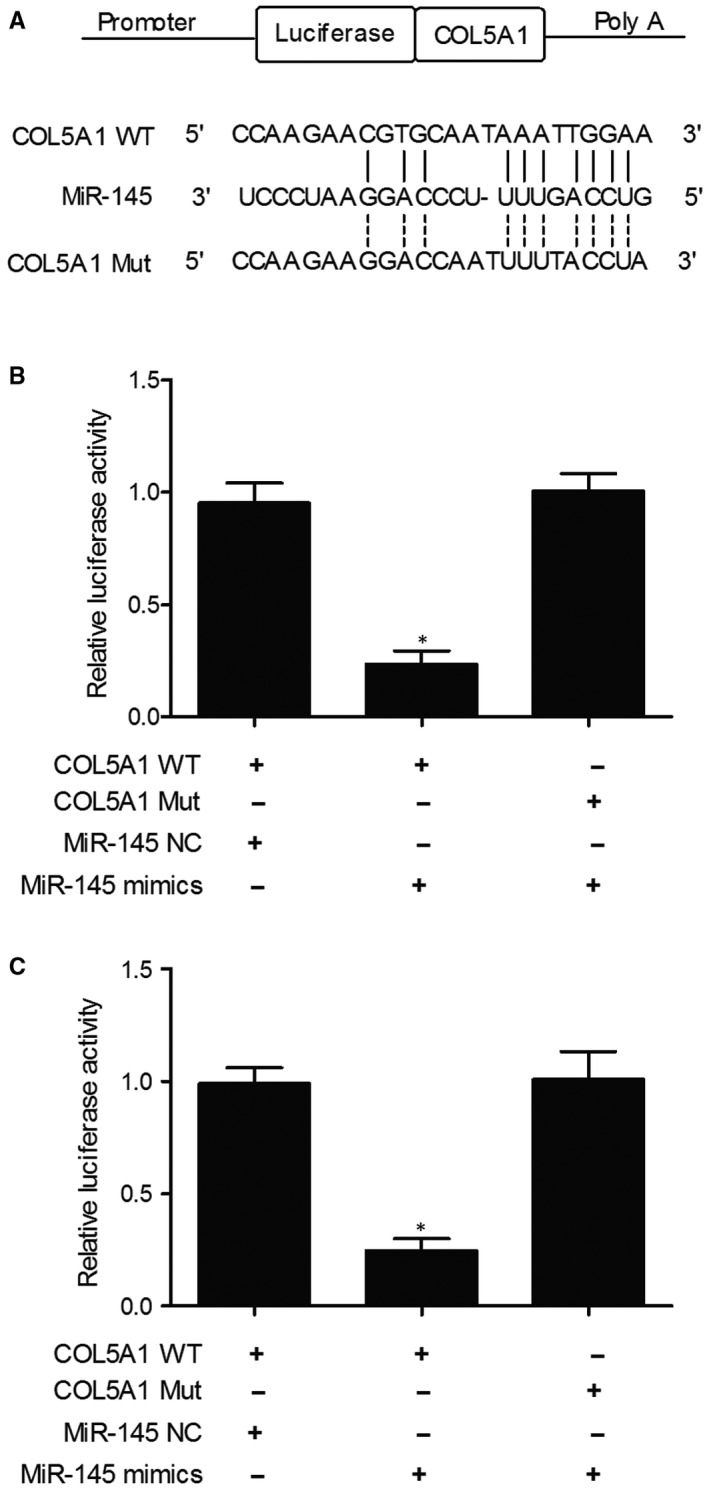
Computational analysis and luciferase assay showed a direct binding between miR‐145 and COL5A1 (*P value < .05. vs COL5A1 + miR‐145 NC group). A, Candidate seed sequences of miR‐145 in the 3′UTR of COL5A1 as detected by computational analysis; B, Luciferase activity of KNS‐89 cells transfected with wild‐type or mutant COL5A1 and miR‐145 mimics or miR‐145 negative controls; C, Luciferase activity of SNB‐19 cells transfected with wild‐type or mutant COL5A1 and miR‐145 mimics or miR‐145 negative controls

Computational analysis of miR‐145 and COL5A1 mRNA has identified COL5A1 mRNA as a potential target of miR‐145. In addition, the relative luciferase activity in KNS‐89 (Figure 4B) and SNB‐19 (Figure 4C) cells co‐transfected with the wild‐type COL5A1 mRNA and miR‐145 mimics was apparently reduced, while the relative luciferase activity in KNS‐89 and SNB‐19 cells co‐transfected with the mutant COL5A1 mRNA and miR‐145 mimics showed no significant difference, suggesting that COL5A1 was a direct target of miR‐145.

### 3.6 MiR‐145 negatively regulated the expression of COL5A1

**FIGURE 5:**
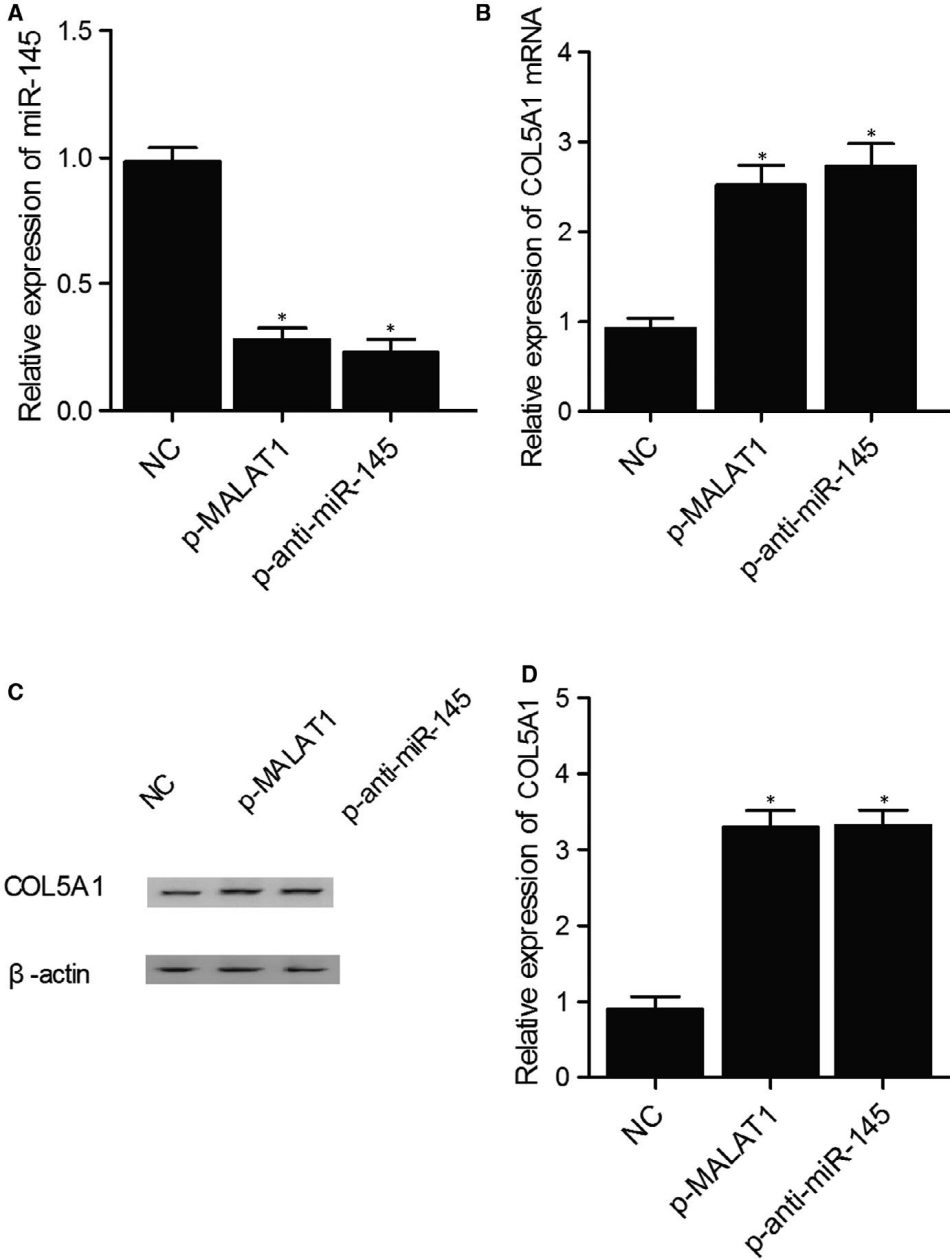
Differentiated expression of miR‐145 and COL5A1 indicated that COL5A1 was negatively regulated by miR‐145 in KNS‐89 cells (*P value < .05. vs NC group). A, Expression of miR‐145 in KNS‐89 cells treated with MALAT1 or anti‐miR‐145; B, Expression of COL5A1 mRNA in KNS‐89 cells treated with MALAT1 or anti‐miR‐145; C, COL5A1 protein expression in KNS‐89 cells treated with MALAT1 or anti‐miR‐145; D, Relative density of COL5A1 protein in KNS‐89 cells treated with MALAT1 or anti‐miR‐145

**FIGURE 6:**
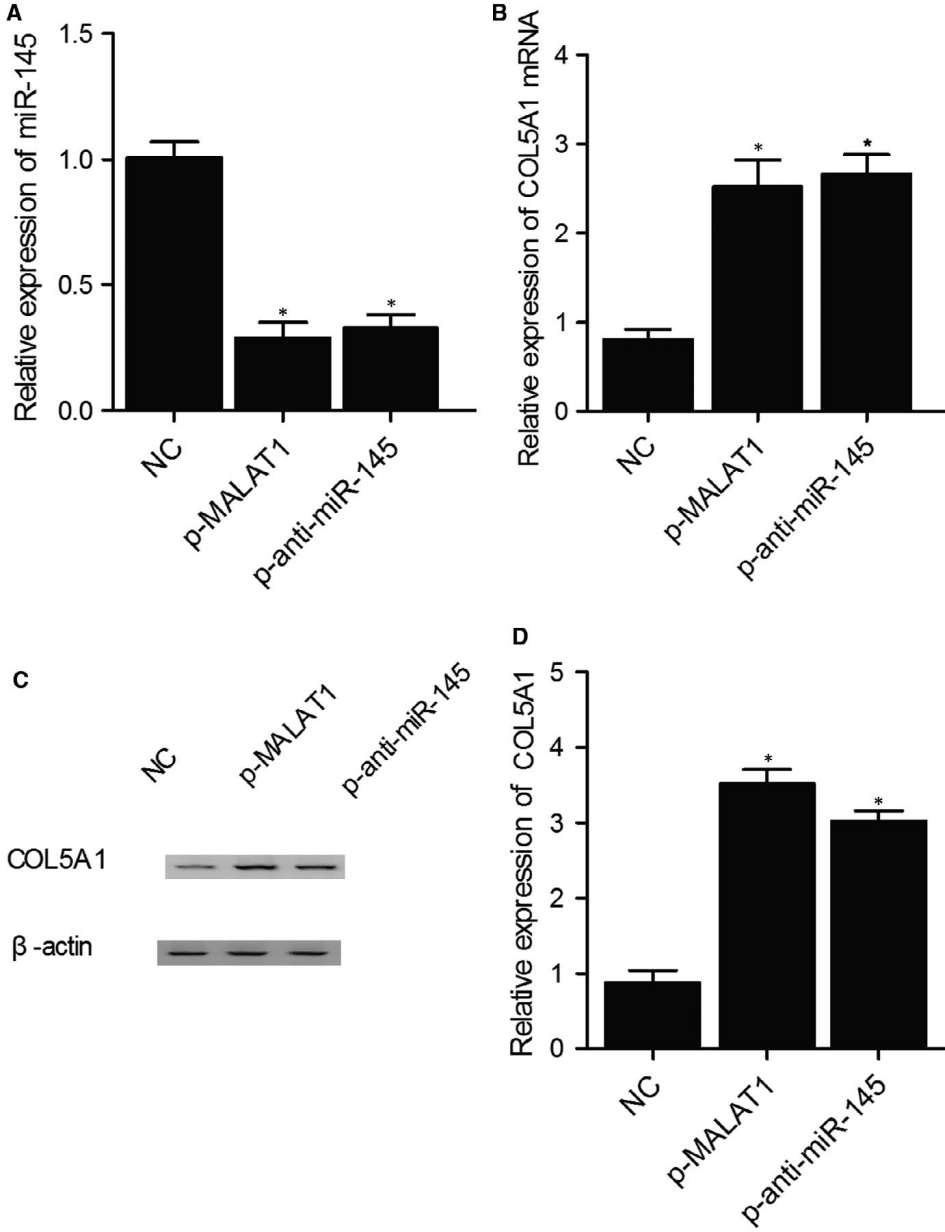
Differentiated expression of miR‐145 and COL5A1 indicated that COL5A1 was negatively regulated by miR‐145 in KNS‐89 cells (*P value < .05. vs NC group). A, Expression of miR‐145 in KNS‐89 cells treated with MALAT1 or anti‐miR‐145; B, Expression of COL5A1 mRNA in KNS‐89 cells treated with MALAT1 or anti‐miR‐145; C, COL5A1 protein expression in KNS‐89 cells treated with MALAT1 or anti‐miR‐145; D, Relative density of COL5A1 protein in KNS‐89 cells treated with MALAT1 or anti‐miR‐145

To further confirm the negative regulatory relationship between miR‐145 and COL5A1, the expression of miR‐145 and COL5A1 mRNA was measured in KNS‐89 and SNB‐19 cells treated with MALAT1 or anti‐miR‐145. As shown in Figure 5 and compared with the negative control, the expression of miR‐145 was significantly inhibited in the KNS‐89 cells treated with MALAT1 or anti‐miR‐145. Meanwhile, the expression of COL5A1 mRNA and protein was significantly elevated in the KNS‐89 cells treated with MALAT1 or anti‐miR‐145. Similar results were also obtained from SNB‐19 cells (Figure 6), indicating that miR‐145 negatively regulated the expression of COL5A1. In summary, the A>G mutation of rs619586 polymorphism in MALAT1 reduced its expression and up‐regulated the expression of miR‐145, which in turn led to the down‐regulation of COL5A1. Since COL5A1 acted as a key factor affecting the invasiveness of meningioma, inhibited COL5A1 expression could reduce the incidence of invasive meningioma.

## 4 DISCUSSION

MALAT1 is an lncRNA with a length of >8000 nt and is located in chromosome 11q13.^12^ MALAT1 is highly conserved in mammals, and previous studies have shown that played a critical role in the tumorigenesis of many cancers. In addition, MALAT1 was shown to participate in a wide range of biological processes, including cell migration, cell proliferation and cell apoptosis.^12^, ^13^, ^14^ It was demonstrated that MALAT1 is a direct target of miR‐145 but may also repress the expression of miR‐145 by directly binding to miR‐145. On the other hand, miR‐145 was shown to suppress the expression of MALAT1 by recruiting MALAT1 for RISC degradation. Furthermore, MALAT1 can modulate TGF‐β1‐induced EndMT (Endothelial‐to‐Mesenchymal Transition) by regulating the expression of miR‐145 as well as TGFBR2 (TGF beta receptor 2) and SMAD3 (Mothers against decapentaplegic homolog 3), the two target genes of miR‐145. The above findings suggested that the MALAT1‐miR‐145‐TGFBR2/SMAD3 signalling axis acts as a key mediator of TGF‐β1‐induced EndMT.^11^ In this study, we confirmed a regulatory relationship between MALAT1 and miR‐145 in meningioma cells using a luciferase reporter assay.

It was demonstrated that miR‐145 was related to high‐grade meningiomas and played an important role in the growth of brain invasive meningioma. Nevertheless, the molecular mechanism underlying the decreased miR‐145 level in anaplastic and atypical meningiomas is still unclear. Interesting, to the location of miR‐145, is in 5q, a region that is conserved in both primary meningiomas and IOMM‐Lee cells. Therefore, promoter methylation may play a role in the silencing of miR‐145 in human malignancies.^15^, ^16^ In a recent study, it was demonstrated in prostate cancer that miR‐145 was transcriptionally inhibited by the methylation of its neighbouring DNA region.^16^ In this study, COL5A1 was predicted as a potential target of miR‐145 via computational analysis. In addition, the luciferase activity of cells co‐transfected with wild‐type COL5A1 and miR‐145 mimics was suppressed compared to that in the cells co‐transfected with mutant COL5A1 and miR‐145 mimics or negative controls, suggesting that COL5A1 acted as a target gene of miR‐145.

The COL5A1 gene is located in 9q34.2‐q34.3 and encodes the proa1(V) chain, a rate‐limiting constituent in the assembly of type V collagen, which in turn is a member of the fibrillar subfamily of collagens.^17^ Type V collagen includes several isoforms, among which a1(V)2a2(V) is the most abundant isoform and is typically complex to type I collagen to create a heterotypic type I/V fibril in tissues including cornea, sclera, bone, tendon and ligament.^18^, ^19^ COL5A1 was demonstrated to act as an essential component of collagen fibrillogenesis. For example, in patients with Ehlers‐Danlos syndrome, COL5A1 mutations were shown to disturb collagen fibrillogenesis.^20^, ^21^ In addition, COL5A1 knockout in mice has resulted in lethal outcomes.^22^ Moreover, COL5A1 may contribute the metastasis of lung adenocarcinoma. Also, some recent studies investigated the relationship between the up‐regulation of COL5A1 in human cancers and invasiveness of meningioma. Specifically, further, the expression of COL5A1 was elevated in muscle‐invasive tumour tissues and metastatic cells in transition cell carcinoma,^23^ and up‐regulated COL5A1 was also identified to contribute to lung adenocarcinoma metastasis by promoting cell invasion and proliferation while suppressing cell apoptosis.^24^ Also, in primary ovarian tumours, the highly expressed COL5A1 was found to be associated with poor prognosis and high risk of metastasis.^25^

Since the SNPs in protein coding genes can affect gene expression and function, they have been implicated in complex diseases.^26^, ^27^ Similarly, SNPs in functional lncRNAs might also alter susceptibility to human diseases. In this study, we found that the rs619586A>G SNP significantly altered PAH risk. In this study, the relative mRNA expression of MALAT1 and miR‐145 was measured in tumour/serum samples genotyped as AA, AG and GG. As the results indicated, the level of MALAT1 decreased in a stepwise fashion with the increasing level of miR‐145 in tumour/serum samples genotyped as AA, AG and GG, respectively. Therefore, the differentiated expression of MALAT1 and miR‐145 indicated a negative correlation between MALAT1 and miR‐145. Furthermore, the expression of COL5A1 mRNA and protein also decreased in a stepwise fashion with the increasing level of miR‐145 in tumour/serum samples genotyped as AA, AG and GG, respectively, suggesting a negative correlation between COL5A1 and miR‐145. All above results indicated that the rs619586A>G SNP in the MALAT1 gene could reduce its expression, thus impairing the effect of miR‐145 on COL5A1. Consistent with previous findings, we observed that the meningioma cells carrying the rs619586G genotype showed a higher expression of COL5A1 than those carrying the AA genotype. Therefore, based on these results, we have been suggested that rs619586A>G SNP conferred protection against meningioma invasion by inhibiting the activation of genes downstream of COL5A1.^28^

## 5 CONCLUSION

In this study, the MALAT1/miR‐145/COL5A1 molecular pathway was established. In particular, with the presence of rs619586 A>G polymorphism, the expression of MALAT1 and COL5A1 was both reduced, leading to reduced invasiveness of meningioma.

## CONFLICT OF INTEREST

None.

## AUTHOR CONTRIBUTION

**Jie Zheng:** Conceptualization (equal); Formal analysis (equal); Investigation (equal); Writing‐original draft (equal). **Chang‐he Pang:** Investigation (equal); Methodology (equal). **Wei Du:** Formal analysis (equal); Investigation (equal); Software (equal). **Lei Wang:** Investigation (equal). **Lai‐guang Sun:** Investigation (equal); Software (equal). **Zhen‐yi Xing:** Project administration (equal); Writing‐original draft (equal); Writing‐review & editing (equal).

## Data Availability

The data that support the findings of this study are available from the corresponding author upon reasonable request.
